# Supplementary material for: Recruitment of two Ndc80 complexes via the CENP-T pathway is sufficient for kinetochore functions
Source: Nat Commun. 2022 Feb 14;13:851. doi: 10.1038/s41467-022-28403-8 (PMC8844409; doi:10.1038/s41467-022-28403-8)
Supplement: Supplementary file 1 — Supplementary Information [file 41467_2022_28403_MOESM1_ESM.pdf]

Recruitment of two Ndc80 complexes via the CENP-T pathway is sufficient for kinetochore functions

Yusuke Takenoshita, Masatoshi Hara, and Tatsuo Fukagawa

Supplementary Information

Supplementary Information contains 7 Supplementary Figures and 2 Tables.

Supplementary Fig. 1

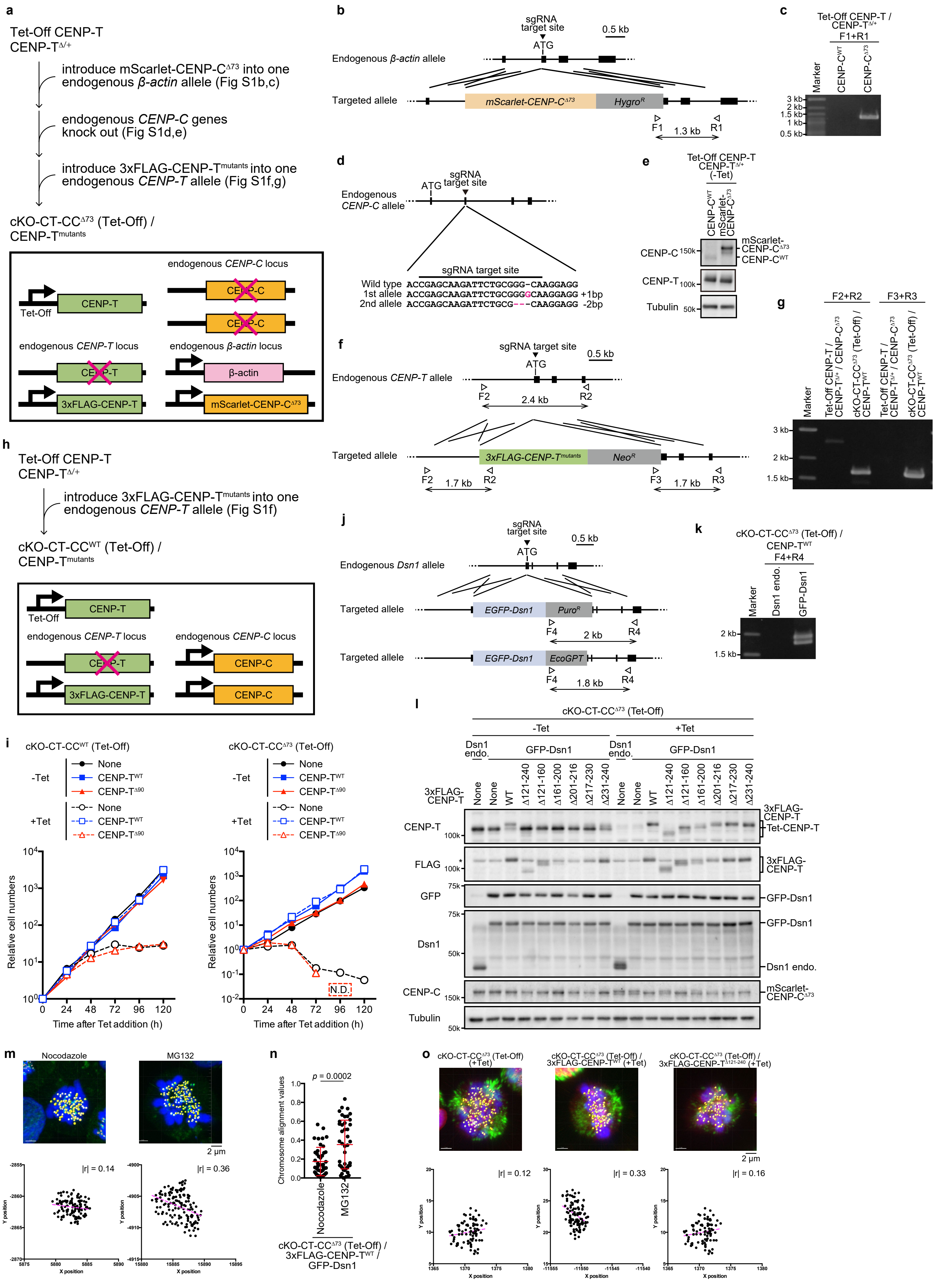

Supplementary Fig. 1 | Strategies for the generation of various CENP-T/CENP-C mutant cell lines. **a,** Generation of cKO-CENP-T cells expressing mScarlet-CENP-$C^{\Delta 73}$ and various 3X FLAG-fused CENP-T mutants. In these cell lines, the endogenous *CENP-C* gene was knocked out (b and c) after mScarlet-CENP-$C^{\Delta 73}$ was introduced into the *β-actin* locus (d and e). Full-length wild-type CENP-T cDNA under the control of Tetracycline responsive promoter (Tet-CENP-T) was integrated at a genome site, and one *CENP-T* gene allele was disrupted. 3X FLAG-fused various CENP-T mutants were introduced into another *CENP-T* allele (f and g). **b,** Map of the chicken *β-actin* locus and targeted integration of mScarlet-CENP-$C^{\Delta 73}$ cDNA into this allele. The indicated primers (F1 and R1) were used for PCR presented in **c**. **c,** PCR profiles confirming target integration of mScarlet-CENP-$C^{\Delta 73}$ cDNA into the *β-actin* locus. **d,** Map of the *CENP-C* locus and sgRNA target sequence for *CENP-C* gene knockout. Mutated sequences in the knockout clones are presented. **e,** Immunoblot analysis confirming that endogenous CENP-C was knocked out, and mScarlet-CENP-$C^{\Delta 73}$ was expressed. **f,** Map of the chicken *CENP-T* locus and targeted integration of various 3X FLAG-fused CENP-T cDNAs into this allele. The indicated primers (F2, F3, R2, and R3) were used for PCR presented in **g**. R2 binds to CENP-T exon 2 and CENP-T cDNA. **g,** PCR profiles confirming target integration of various 3X FLAG-fused CENP-T cDNAs into the endogenous *CENP-T* locus. **h,** Generation of cKO-CENP-T cells expressing 3X FLAG-fused various CENP-T mutants. The same strategy presented in **a** was applied, albeit the *CENP-C* gene was not manipulated. **i,** The growth of cKO-CENP-T cells in either the presence (left: CENP-C^{WT}) or absence (right: CENP-$C^{\Delta 73}$) of the CENP-C-Mis12C interaction expressing either 3X FLAG-fused CENP-T^{WT} or CENP-$T^{\Delta 90}$ in the absence or presence or Tet. **j,** Map of the chicken *Dsn1* locus and targeted integration of GFP-Dsn1 cDNA into this allele. The indicated primers (F4 and R4) were used for PCR presented in **k**. F4 binds to PGK promoter in drug resistance gene cassettes. **k,** PCR profiles confirming target integration of GFP-Dsn1 into the *Dsn1* locus. **l,** Immunoblot analysis confirming the expression of GFP-Dsn1, various 3X FLAG-fused CENP-T mutants, and mScarlet-CENP-$C^{\Delta 73}$. The cells were cultured in either the absence or presence of Tet for 30 h. Asterisks indicate non-specific bands. **m,** Comparison of chromosome alignment in cKO CT-$CC^{\Delta 73}$/3X FLAG-fused CENP-T^{WT}/GFP-Dsn1 cells treated Nocodazole (absence of microtubule) or MG132 (presence of microtubules). Positions of each kinetochore signal are plotted and a linear approximation straight-line is drawn. Absolute values of a correlation coefficient are defined as chromosome alignment values. **n,** Chromosome alignment values of images in **m**. Error bars indicate the mean and standard deviation. *p* values were calculated by two-tailed Welch's t-test. **o,** Typical images of mitotic chromosomes and chromosome alignment values in cKO-CENP-T/CENP-$C^{\Delta 73}$, cKO-CENP-T/CENP-$C^{\Delta 73}$ expressing CENP-T^{WT}, or CENP-$T^{\Delta 121\text{-}240}$.

Supplementary Fig. 2

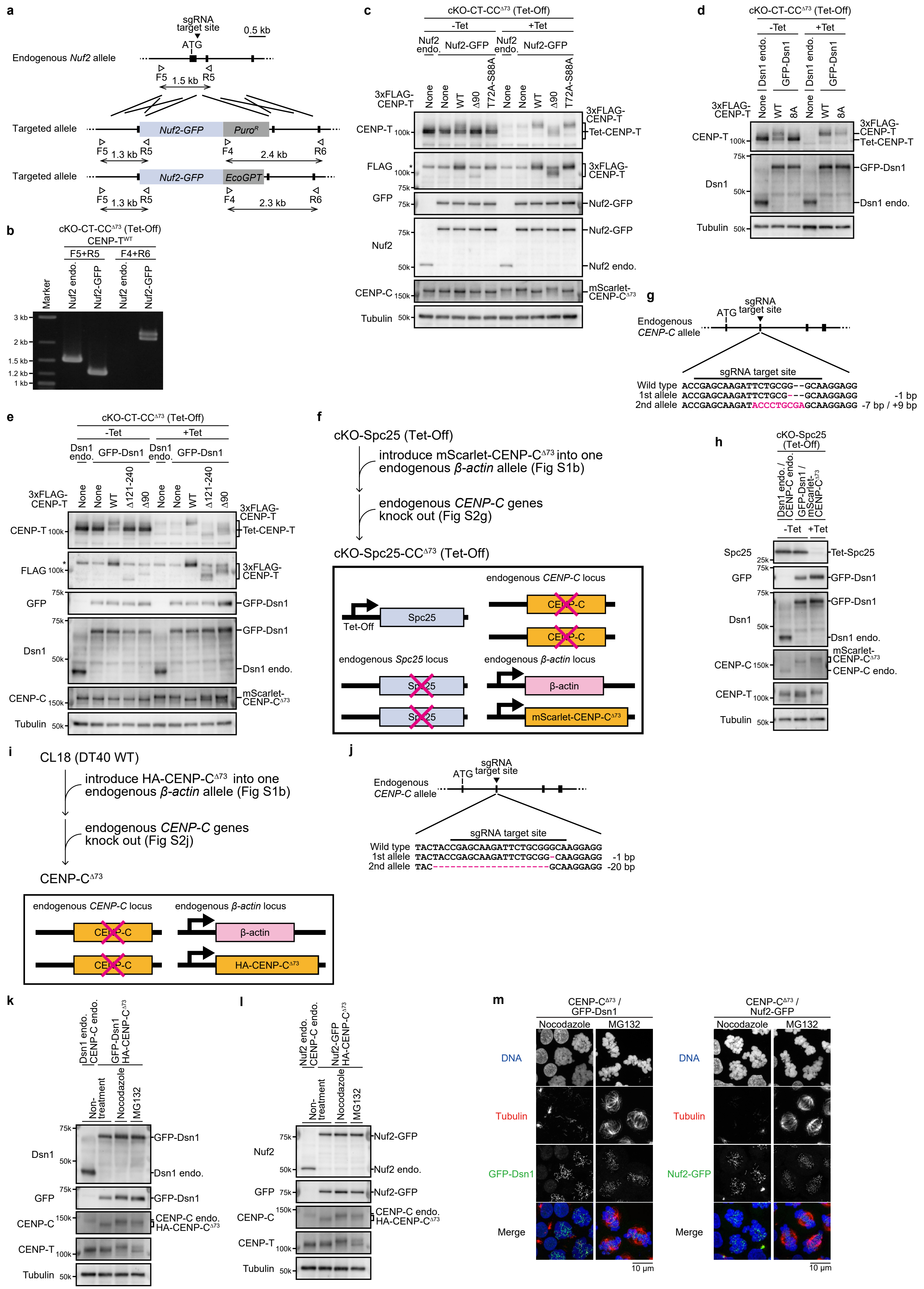

Supplementary Fig. 2 | Expression levels of Nuf2-GFP, GFP-Dsn1, or various CENP-T mutants. a, Map of the chicken *Nuf2* locus and targeted integration of Nuf2-GFP cDNA into this allele. The indicated primers (F4, F5, R5, and R6) were used for PCR presented in **b**. R5 binds to Nuf2 exon 2 and Nuf2 cDNA. F4 binds to PGK promoter in drug resistance gene cassettes. **b,** PCR profiles confirming target integration of Nuf2-GFP into the *Nuf2* locus. **c,** Immunoblot analysis confirming the expression of Nuf2-GFP and various CENP-T mutants. The cells were cultured in either the absence or presence of Tet for 30 h. CENP-C migration profiles were slightly different, due to CENP-C phosphorylation at mitosis, when mutant CENP-T was expressed. Asterisks indicate non-specific bands. **d,** Immunoblot analysis confirming the expression of GFP-Dsn1 and CENP-T^{8A} mutant. The cells were cultured in either the absence or presence of Tet for 48 h. **e,** Immunoblot analysis confirming the expression of various 3X FLAG-CENP-T mutants, GFP-Dsn1, and mScarlet-CENP-$C^{\Delta 73}$ in cKO-CENP-T cells expressing CENP-$C^{\Delta 73}$ in either the absence or presence of Tet for 30 h. Asterisks indicate non-specific bands. **f,** Generation of cKO-Spc25 cells expressing CENP-$C^{\Delta 73}$. In this cell line, the endogenous *CENP-C* gene was knocked out, after mScarlet-CENP-$C^{\Delta 73}$ was introduced into the *β-actin* locus (Supplementary Fig. 1b). Full-length wild-type Spc25 cDNA under the control of Tetracycline responsive promoter was integrated at a genome site, and two Spc25 gene alleles were disrupted. **g,** Map of the *CENP-C* locus and sgRNA target sequence for *CENP-C* gene knockout. Mutated sequences in cKO-Spc25/CENP-$C^{\Delta 73}$ cells are presented. **h,** Immunoblot analysis confirming the expression of mScarlet-CENP-$C^{\Delta 73}$ and GFP-Dsn1 in cKO-Spc25/CENP-$C^{\Delta 73}$ cells expressing GFP-Dsn1. The cells were cultured in either the absence or presence of Tet for 18 h. Spc25 was not detected in these cells after Tet addition. **i,** Generation of CENP-$C^{\Delta 73}$ cells. In this cell line, the endogenous *CENP-C* gene was knocked out after HA-fused CENP-$C^{\Delta 73}$ was introduced into the *β-actin* locus. **j,** Map of the *CENP-C* locus and sgRNA target sequence for *CENP-C* gene knockout. Mutated sequences in CENP-$C^{\Delta 73}$ cells are presented. **k,** Immunoblot analysis confirming the expression of HA-CENP-$C^{\Delta 73}$ and GFP-Dsn1 in CENP-$C^{\Delta 73}$ cells expressing GFP-Dsn1. **l,** Immunoblot analysis confirming the expression of HA-CENP-$C^{\Delta 73}$ and Nuf2-GFP in CENP-$C^{\Delta 73}$ cells expressing Nuf2-GFP. **m,** Images of mitotic chromosomes (blue) and microtubules staining (red) in CENP-$C^{\Delta 73}$/GFP-Dsn1 cells.

Supplementary Fig. 3

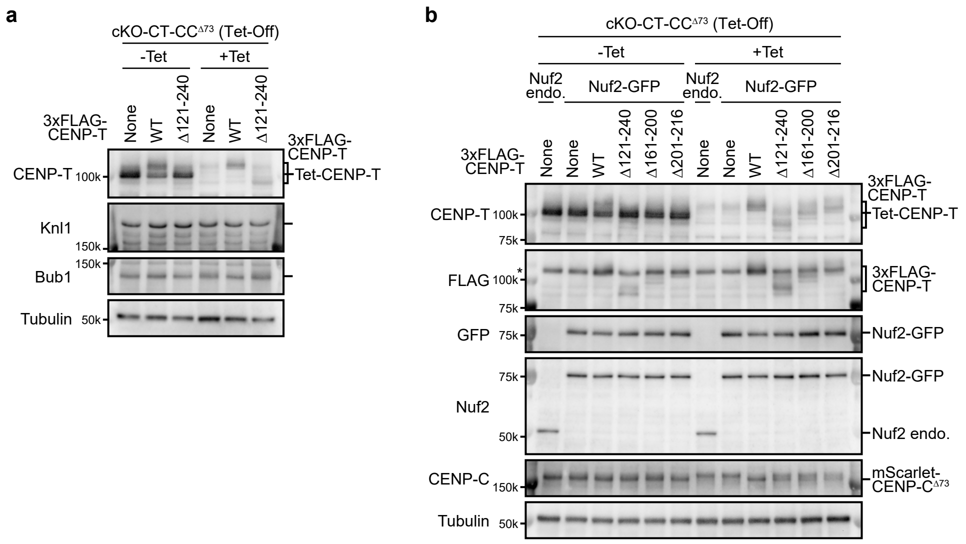

Supplementary Fig. 3 | Expression levels of Nuf2-GFP, Knl1, and Bub1 in cKO-CENP-T cells in the absence of the CENP-C-Mis12C interaction expressing various CENP-T mutants. a, Immunoblot analysis for Bub1 and Knl1 in the cKO-CT-CC$^{\Delta 73}$ cells expressing CENP-T$^{\Delta 121\text{-}240}$. The cells were cultured in either the absence or presence of Tet for 30 h. **b,** Immunoblot analysis confirming the expression of various CENP-T mutants and Nuf2-GFP in cKO-CENP-T cells expressing mScarlet-CENP-C$^{\Delta 73}$ and various CENP-T mutants. The cells were cultured in either the absence or presence of Tet for 30 h. Asterisks indicate non-specific bands.

Supplementary Fig. 4

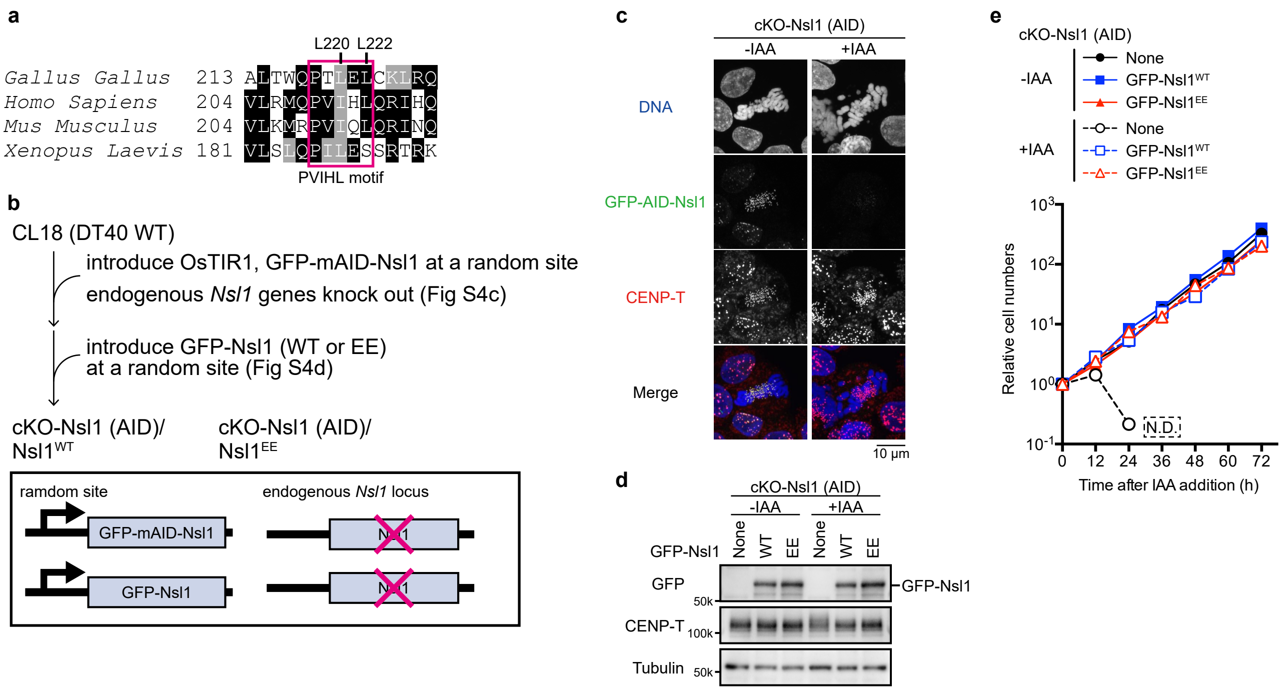

Supplementary Fig. 4 | Characterization of cKO-Nsl1 DT40 cells. a, Amino acids alignment of Nsl1 around the PVIHL motif between various species. The PVIHL motif in human Nsl1 is predicted as a critical region for Ndc80C-Mis12C interaction. Leucine 220 (L220) and leucine 222 (L222) residues of $Nsl1^{WT}$ were substituted with glutamic acid in $Nsl1^{EE}$**. b,** Schematic diagram for generation of AID-based cKO-Nsl1 DT40 cells. GFP-$Nsl1^{WT}$ or GFP-$Nsl1^{EE}$ was introduced into cKO-Nsl1 cells. **c,** Detection of GFP-AID-Nsl1 in cKO-Nsl1 cells either in the absence or presence of IAA. DNA was stained with DAPI. CENP-T was stained using an anti-CENP-T antibody (red). **d,** Immunoblot analysis confirming the expression of GFP-$Nsl1^{WT}$ or GFP-$Nsl1^{EE}$ in AID-based cKO-Nsl1 cells expressing GFP-$Nsl1^{WT}$ or GFP-$Nsl1^{EE}$. The cells were cultured in either the absence or presence of IAA for 6 h. **e,** The growth of cKO-Nsl1 cells expressing either GFP-$Nsl1^{WT}$ or GFP-$Nsl1^{EE}$ in the absence or presence of IAA.

Supplementary Fig. 5

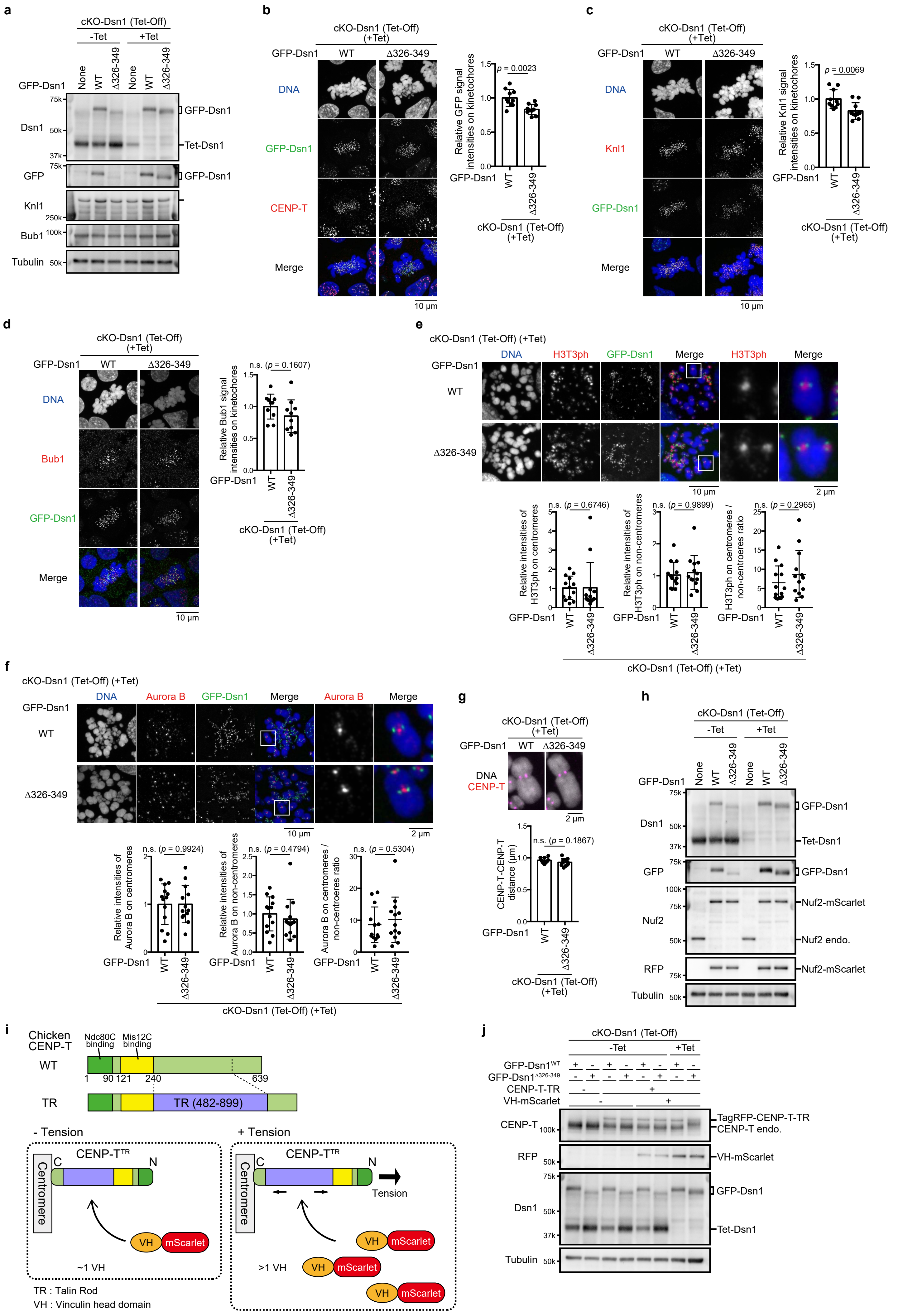

Supplementary Fig. 5 | Characterization of cKO-Dsn1 cells expressing Dsn1$^{\Delta 326\text{-}349}$. a, Immunoblot analysis confirming expression of the GFP-Dsn1^{WT} or Dsn1$^{\Delta 326\text{-}349}$ in cKO-Dsn1 cells expressing GFP-fused Dsn1^{WT} or Dsn1$^{\Delta 326\text{-}349}$. GFP-Dsn1^{WT} or GFP-Dsn1$^{\Delta 326\text{-}349}$ were introduced into the endogenous locus as presented in Supplementary Fig. 1j. The cells were cultured in either the absence or presence of Tet for 30 h. **b,** GFP-Dsn1 levels at kinetochores in cKO-Dsn1 cells expressing either GFP-Dsn1^{WT} or GFP-Dsn1$^{\Delta 326\text{-}349}$ in the presence of Tet for 30 h. CENP-T was stained using an anti-CENP-T antibody (red). DNA was stained with DAPI. Error bars indicate the mean and standard deviation. *p* values were calculated by two-tailed Welch's t-test. **c,** Knl1C levels at kinetochores in cKO-Dsn1 cells expressing either GFP-Dsn1^{WT} or GFP-Dsn1$^{\Delta 326\text{-}349}$ in the presence of Tet for 30 h. Knl1 was stained using an anti-Knl1 antibody (red). DNA was stained with DAPI. Error bars show the mean and standard deviation. *p* values were calculated as in **b**. **d,** Bub1 levels at kinetochores in cKO-Dsn1 cells expressing either GFP-Dsn1^{WT} or GFP-Dsn1$^{\Delta 326\text{-}349}$ in the presence of Tet for 30 h. Bub1 was stained using an anti-Bub1 antibody (red). DNA was stained with DAPI. Error bars indicate the mean and standard deviation. *p* values were calculated as in **b**. **e,** H3T3ph staining profiles on chromosomes in cKO-Dsn1 cells expressing either GFP-Dsn1^{WT} or GFP-Dsn1$^{\Delta 326\text{-}349}$ in the presence of Tet for 30 h. H3T3ph was stained using an anti-H3T3ph antibody (red). DNA was stained with DAPI. The insets present magnified views of the boxed region. Graphs summarize H3T3ph localization profiles on centromeres or non-centromere regions. Error bars indicate the mean and standard deviation. *p* values were calculated as in **b**. **f,** Aurora B staining profile on entire chromosomes in cKO-Dsn1 cells expressing either GFP-Dsn1^{WT} or GFP-Dsn1$^{\Delta 326\text{-}349}$ in the presence of Tet for 30 h. Aurora B was stained using an anti-Aurora B antibody (red). DNA was stained with DAPI. The insets present magnified views of the boxed region. Graphs summarize Aurora B localization profiles on centromeres or non-centromere regions. Error bars show the mean and standard deviation. *p* values were calculated as in **b**. **g,** Kinetochore-kinetochore distances of a mitotic chromosome in cKO-Dsn1 cells expressing either GFP-Dsn1^{WT} or GFP-Dsn1$^{\Delta 326\text{-}349}$ in the presence of Tet for 30 h. CENP-T was stained using an anti-CENP-T antibody (red). DNA was stained with DAPI. Error bars indicate the mean and standard deviation. *p* values were calculated as in **b**. **h,** Immunoblot analysis confirming the expression of Nuf2-mScarlet in cKO-Dsn1 cells expressing either GFP-Dsn1^{WT} or GFP-Dsn1$^{\Delta 326\text{-}349}$. **i,** Schematic representation of a tension sensor system using Talin Rod (TR)-CENP-T (CENP-T-TR) and Vinculin head (VH) domain. When the tension was applied to CENP-T-TR, the Vinculin-binding domain was exposed, causing VH binding. Using mScarlet-VH, force applied to TR was visualized as kinetochore signals on TR. **j,** Immunoblot analysis confirming expression of CENP-T-TR in cKO-Dsn1 cells expressing either GFP-Dsn1^{WT} or GFP-Dsn1$^{\Delta 326\text{-}349}$.

Supplementary Fig. 6

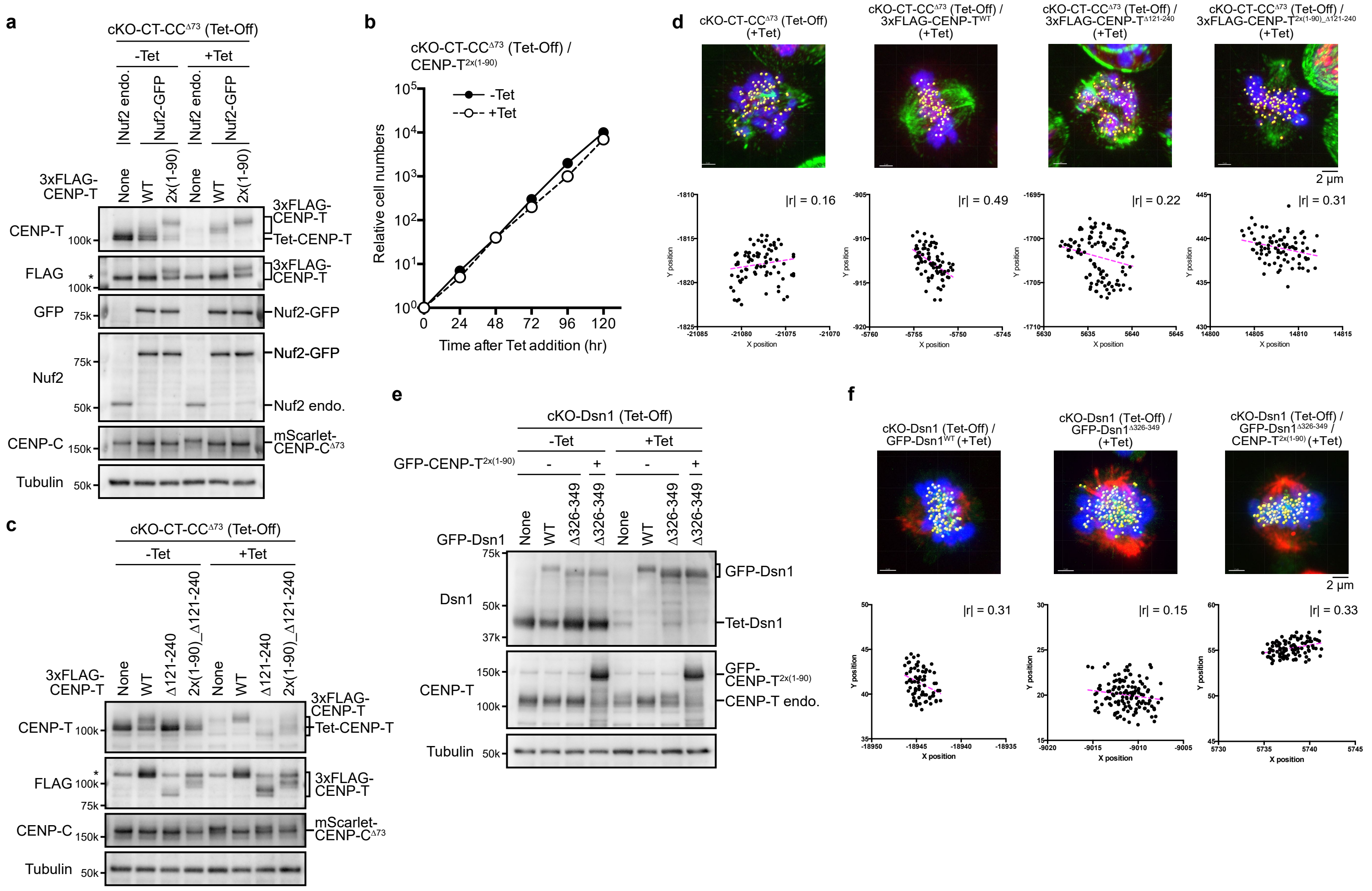

Supplementary Fig. 6 | Characterization of cKO-CENP-T expressing CENP-T$^{2X(1-90)}$. a, Expression of 3X FLAG-fused CENP-T$^{2X(1-90)}$ and Nuf2-GFP in cKO-CENP-T cells expressing CENP-C$^{\Delta73}$, 3X FLAG-CENP-T$^{2X(1-90)}$ and Nuf2-GFP in either the absence or presence of Tet for 30 h. Asterisks indicate non-specific bands. **b,** The growth of cKO-CENP-T cells expressing mScarlet-CENP-C$^{\Delta73}$ and 3X FLAG-fused CENP-T$^{2X(1-90)}$ in either the absence or presence of Tet. **c,** Expression levels of 3X FLAG-fused CENP-T$^{2X(1-90)_\Delta121-240}$ in cKO-CENP-T cells expressing mScarlet-CENP-C$^{\Delta73}$ and CENP-T$^{2X(1-90)_\Delta121-240}$ in either the absence or presence of Tet for 30 h. Asterisks indicate non-specific bands. **d,** Mitotic chromosome images to evaluate chromosome alignment in cKO-CENP-T/CENP-C$^{\Delta73}$, cKO-CENP-T/CENP-C$^{\Delta73}$ expressing CENP-T^{WT}, CENP-T$^{\Delta121-240}$, or CENP-T$^{2X(1-90)_\Delta121-240}$. **e**, Expression levels of CENP-T$^{2X(1-90)}$ in cKO-Dsn1 cells expressing either GFP-Dsn1^{WT} or GFP-Dsn1$^{\Delta326-349}$ in either the absence or presence of Tet for 30 h. **f**, Mitotic chromosome images to evaluate chromosome alignment in cKO-Dsn1 cells expressing Dsn1^{WT}, Dsn1$^{\Delta326-349}$, or both Dsn1$^{\Delta326-349}$ and CENP-T$^{2X(1-90)}$.

Supplementary Fig. 7

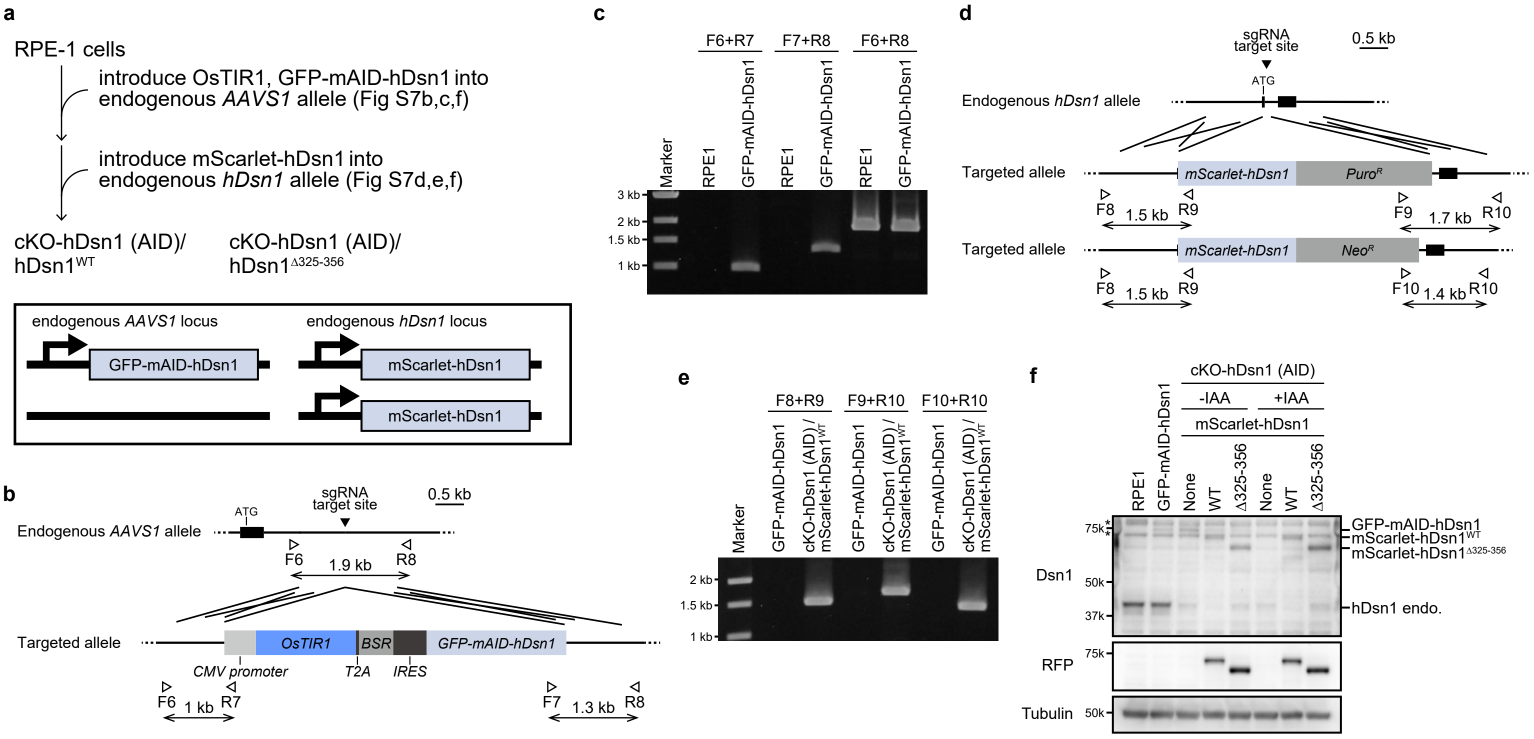

Supplementary Fig. 7 | Generation of cKO-hDsn1 RPE1 cells expressing hDsn1$^{325\text{-}356}$. a, Generation of AID-based cKO-hDsn1 RPE-1 cells (cKO-hDsn1 (AID)). The GFP-AID-hDsn1 construct containing OsTIR1 was targeted into the human *AAVS1* locus (**b**, **c**, and **f**). Then, mScarlet-fused hDsn1^{WT} or hDsn1$^{\Delta 325\text{-}356}$ were integrated into the endogenous *Dsn1* locus (**d**, **e**, and **f**). When IAA was added to these cells, GFP-AID-hDsn1 was degraded, but hDsn1^{WT} or hDsn1$^{\Delta 325\text{-}356}$ was still expressed. **b,** Map of the human *AAVS1* locus and targeted integration of GFP-AID-hDsn1 construct containing OsTIR1 into this locus. The indicated primers (F6, F7, R7, and R8) were used for PCR presented in **c**. **c,** PCR profiles confirming target integration of GFP-AID-hDsn1 construct into the *AAVS1* locus. **d,** Map of the *hDsn1* locus and targeted integration of mScarlet-fused hDsn1^{WT} or hDsn1$^{\Delta 325\text{-}356}$ into this locus. The indicated primers (F8, F9, F10, R9, and R10) were used for PCR presented in **e**. **e,** PCR profiles confirming target integration of mScarlet-fused hDsn1 into the *hDsn1* locus. **f,** Immunoblot analysis confirming the expression of hDsn1 with an anti-hDsn1 antibody in cKO-hDsn1 (AID) cells expressing hDsn1^{WT} or hDsn1$^{\Delta 325\text{-}356}$ in either the absence or presence of IAA for 2 h. Asterisks indicate non-specific bands.

Supplymentary Table 1. A list of cell lines established in this study

cell line
cKO-CT-CC^{WT}
cKO-CT-CC^{WT} / CENP-T^{WT}
cKO-CT-CC^{WT} / CENP-T$^{\Delta 90}$
cKO-CT-CC^{WT} / CENP-T$^{\Delta 121\text{-}240}$
cKO-CT-CC$^{\Delta 73}$
cKO-CT-CC$^{\Delta 73}$ / CENP-T$^{\Delta 90}$
cKO-CT-CC$^{\Delta 73}$ / CENP-T$^{\Delta 121\text{-}240}$
cKO-CT-CC$^{\Delta 73}$ / CENP-T$^{\Delta 121\text{-}160}$
cKO-CT-CC$^{\Delta 73}$ / CENP-T$^{\Delta 161\text{-}200}$
cKO-CT-CC$^{\Delta 73}$ / CENP-T$^{\Delta 201\text{-}216}$
cKO-CT-CC$^{\Delta 73}$ / CENP-T$^{\Delta 217\text{-}230}$
cKO-CT-CC$^{\Delta 73}$ / CENP-T$^{\Delta 231\text{-}240}$
cKO-CT-CC$^{\Delta 73}$ / GFP-Dsn1
cKO-CT-CC$^{\Delta 73}$ / CENP-T$^{\Delta 121\text{-}240}$ / GFP-Dsn1
cKO-CT-CC$^{\Delta 73}$ / CENP-T$^{\Delta 121\text{-}160}$ / GFP-Dsn1
cKO-CT-CC$^{\Delta 73}$ / CENP-T$^{\Delta 161\text{-}200}$ / GFP-Dsn1
cKO-CT-CC$^{\Delta 73}$ / CENP-T$^{\Delta 201\text{-}216}$ / GFP-Dsn1
cKO-CT-CC$^{\Delta 73}$ / CENP-T$^{\Delta 217\text{-}230}$ / GFP-Dsn1
cKO-CT-CC$^{\Delta 73}$ / CENP-T$^{\Delta 231\text{-}240}$ / GFP-Dsn1
cKO-CT-CC$^{\Delta 73}$ / Nuf2-GFP
cKO-CT-CC$^{\Delta 73}$ / CENP-T^{WT} / Nuf2-GFP
cKO-CT-CC$^{\Delta 73}$ / CENP-T$^{\Delta 90}$ / Nuf2-GFP
cKO-CT-CC$^{\Delta 73}$ / CENP-T$^{T72A\text{-}S88A}$ / Nuf2-GFP
cKO-CT-CC$^{\Delta 73}$ / CENP-T^{8A}
cKO-CT-CC$^{\Delta 73}$ / CENP-T^{8A} / GFP-Dsn1
cKO-CT-CC$^{\Delta 73}$ / CENP-T$^{\Delta 90}$ / GFP-Dsn1
cKO-Spc25-CC$^{\Delta 73}$ / GFP-Dsn1
CENP-C$^{\Delta 73}$ / GFP-Dsn1
CENP-C$^{\Delta 73}$ / Nuf2-GFP
cKO-CT-CC$^{\Delta 73}$ / CENP-T$^{\Delta 121\text{-}240}$ / Nuf2-GFP
cKO-CT-CC$^{\Delta 73}$ / CENP-T$^{\Delta 161\text{-}200}$ / Nuf2-GFP
cKO-CT-CC$^{\Delta 73}$ / CENP-T$^{\Delta 201\text{-}216}$ / Nuf2-GFP
cKO-Nsl1
cKO-Nsl1 / Nsl1^{WT}
cKO-Nsl1 / Nsl1^{EE}
cKO-Dsn1 / Dsn1^{WT}
cKO-Dsn1 / Dsn1^{WT} / Nuf2-mScarlet
cKO-Dsn1 / Dsn1$^{\Delta 326\text{-}349}$
cKO-Dsn1 / Dsn1$^{\Delta 326\text{-}349}$ / Nuf2-mScarlet
cKO-Dsn1 / Dsn1^{WT} / CENP-T-TR / VH-mScarlet
cKO-Dsn1 / Dsn1$^{\Delta 326\text{-}349}$ / CENP-T-TR / VH-mScarlet
cKO-CT-CC$^{\Delta 73}$ / CENP-T$^{2x(1\text{-}90)}$
cKO-CT-CC$^{\Delta 73}$ / CENP-T$^{2x(1\text{-}90)}$ / Nuf2-GFP
cKO-CT-CC$^{\Delta 73}$ / CENP-T$^{2x(1\text{-}90)_\Delta 121\text{-}240}$
cKO-Dsn1 / Dsn1$^{\Delta 326\text{-}349}$ / CENP-T$^{2x(1\text{-}90)}$
cKO-hDsn1
cKO-hDsn1 / Dsn1^{WT}
cKO-hDsn1 / Dsn1$^{\Delta 325\text{-}356}$

Supplymentary Table 2. A list of primers used in genotyping

Primer#	Primer sequence (5' -> 3')
F1	CTTGACTGACTGAGATACAG
F2	GGCGTAGGCCAGAGGTGTCTCC
F3	GCCGATCATAATCAGCCATACC
F4	ATTCCGCACGCTTCAAAAGC
F5	GGCATCTAGGAACGTTAAACC
F6	TGCCGTCTCTCTCCTGAGTC
F7	ATGGAGTTGGTGATGGATGAAC
F8	CAGGTAGAAGGACAGATATGTGC
F9	GGCTCGGCTTCACCGTCACC
F10	GGCTACCCGTGATATTGCTGAAG
R1	ACATTTGTCCCTAGATAAGAGG
R2	AGGCTCTGGTCGAGCATCTTCTG
R3	AAATCCTGCTCCTCGATTCCAGC
R4	CTCTCCAGGGTCAGGTTCTGTG
R5	CCAGGCGGATTCCATACACC
R6	CAAGTAGTGGAACTGCCAAC
R7	GAAAGTCCCTATTGGCGTTAC
R8	TACCCCGAAGAGTGAGTTTGC
R9	TGAAGGCCCTGGAGCCGTACATG
R10	GTCACCCAGGATAGATTGAGTGG
