# Supplementary material for: Recruitment of two Ndc80 complexes via the CENP-T pathway is sufficient for kinetochore functions
Source: Nat Commun. 2022 Feb 14;13:851. doi: 10.1038/s41467-022-28403-8 (PMC8844409; doi:10.1038/s41467-022-28403-8)
Supplement: Supplementary file 3 — Reporting Summary [file 41467_2022_28403_MOESM3_ESM.pdf]

nature portfolio

Corresponding author(s): Tatsuo Fukagawa

Last updated by author(s): Jan 7, 2022

Reporting Summary

Nature Portfolio wishes to improve the reproducibility of the work that we publish. This form provides structure for consistency and transparency in reporting. For further information on Nature Portfolio policies, see our Editorial Policies and the Editorial Policy Checklist.

Statistics

For all statistical analyses, confirm that the following items are present in the figure legend, table legend, main text, or Methods section.

n/a	Confirmed	
☐	☒	The exact sample size (*n*) for each experimental group/condition, given as a discrete number and unit of measurement
☐	☒	A statement on whether measurements were taken from distinct samples or whether the same sample was measured repeatedly
☐	☒	The statistical test(s) used AND whether they are one- or two-sided<br>*Only common tests should be described solely by name; describe more complex techniques in the Methods section.*
☒	☐	A description of all covariates tested
☒	☐	A description of any assumptions or corrections, such as tests of normality and adjustment for multiple comparisons
☐	☒	A full description of the statistical parameters including central tendency (e.g. means) or other basic estimates (e.g. regression coefficient) AND variation (e.g. standard deviation) or associated estimates of uncertainty (e.g. confidence intervals)
☐	☒	For null hypothesis testing, the test statistic (e.g. *F*, *t*, *r*) with confidence intervals, effect sizes, degrees of freedom and *P* value noted<br>*Give P values as exact values whenever suitable.*
☒	☐	For Bayesian analysis, information on the choice of priors and Markov chain Monte Carlo settings
☒	☐	For hierarchical and complex designs, identification of the appropriate level for tests and full reporting of outcomes
☒	☐	Estimates of effect sizes (e.g. Cohen's *d*, Pearson's *r*), indicating how they were calculated

Our web collection on statistics for biologists contains articles on many of the points above.

Software and code

Policy information about availability of computer code

Data collection	NIS-elements v4.60 (Nikon), image lab touch software v2.3.0.07 (Bio-Rad), guava soft 3.1.1 (Merk)
Data analysis	Image Lab v5.2.1 (Bio-Rad), GraphPad Prism7 (GraphPad Software), Imaris v9.0.2. (Bitplane), Fiji v1.53, Adobe Photoshop v23.1.0 (Adobe), CRISPOR v4.99 (PMID: 29762716)

For manuscripts utilizing custom algorithms or software that are central to the research but not yet described in published literature, software must be made available to editors and reviewers. We strongly encourage code deposition in a community repository (e.g. GitHub). See the Nature Portfolio guidelines for submitting code & software for further information.

Data

Policy information about availability of data

All manuscripts must include a data availability statement. This statement should provide the following information, where applicable:

- Accession codes, unique identifiers, or web links for publicly available datasets
- A description of any restrictions on data availability
- For clinical datasets or third party data, please ensure that the statement adheres to our policy

All of source data have been provided as Source data.

Field-specific reporting

Please select the one below that is the best fit for your research. If you are not sure, read the appropriate sections before making your selection.

☒ Life sciences ☐ Behavioural & social sciences ☐ Ecological, evolutionary & environmental sciences

For a reference copy of the document with all sections, see nature.com/documents/nr-reporting-summary-flat.pdf

Life sciences study design

All studies must disclose on these points even when the disclosure is negative.

Sample size	No statistical method was used for sample size predetermination. The sample size was chosen based on other studies with similar methodologies (PMID: 33207191, 30420662, 31676716). Since previous studies drew reasonable conclusions, sample size we used in this study is sufficient to draw a conclusion.
Data exclusions	No data were excluded.
Replication	All experiments were successfully replicated, and the number of independent experiments were specified in the Methods (Statistics and Reproducibility).
Randomization	All samples were assigned randomly into experimental groups. Microscopy image acquisition was performed randomly.
Blinding	No blinding. Blinding was technically difficult because experiments and analysis were carried out by the same investigator.

Reporting for specific materials, systems and methods

We require information from authors about some types of materials, experimental systems and methods used in many studies. Here, indicate whether each material, system or method listed is relevant to your study. If you are not sure if a list item applies to your research, read the appropriate section before selecting a response.

Materials & experimental systems

n/a	Involved in the study	
☐	☒	Antibodies
☐	☒	Eukaryotic cell lines
☒	☐	Palaeontology and archaeology
☒	☐	Animals and other organisms
☒	☐	Human research participants
☒	☐	Clinical data
☒	☐	Dual use research of concern

Methods

n/a	Involved in the study	
☒	☐	ChIP-seq
☐	☒	Flow cytometry
☒	☐	MRI-based neuroimaging

Antibodies

Antibodies used	Rabbit polyclonal anti-chicken CENP-T (Hori et al., 2008, Fukagawa Lab, Osaka university); Rabbit polyclonal anti-chicken CENP-C (Fukagawa et al., 1999, in this study, Fukagawa Lab, Osaka university); Rabbit polyclonal anti-chicken Dsn1 (Hara et al., 2018, Fukagawa Lab, Osaka university); Rabbit polyclonal anti-chicken Nuf2 (Hori et al., 2003, Fukagawa Lab, Osaka university); Rabbit polyclonal anti-chicken Ndc80 (Hori et al., 2003, Fukagawa Lab, Osaka university); Rabbit polyclonal anti-chicken Spc25 (in this study, Fukagawa Lab, Osaka university); Rabbit polyclonal anti-chicken Knl1 (Hori et al., 2013, Fukagawa Lab, Osaka university); Rabbit anti polyclonal-chicken Mis12 (Kline et al., 2006, Fukagawa Lab, Osaka university); Rabbit polyclonal anti-chicken Bub1 (Hori et al., 2013, Fukagawa Lab, Osaka university); Rabbit polyclonal anti-chicken Aurora B (Hori et al., 2013, Fukagawa Lab, Osaka university); Mouse monoclonal anti-H3T3ph (Hori et al., 2013, Kimura Lab, Tokyo Institute of Technology); Mouse monoclonal anti-human Ndc80 (abcam, Cat.no. ab3613); Rabbit polyclonal anti-human Dsn1 (Bio Academia, Cat.no. 70-101); Rabbit polyclonal anti-GFP (MBL, Cat.no. 598); Mouse monoclonal anti-FLAG M2 (Sigma, Cat.no. F1804); Rat monoclonal anti-RFP (Chromotek, Cat.no. 5f8); Mouse anti-α-tubulin (Sigma, Cat.no. T9026); FITC-conjugated mouse anti-α-tubulin (Sigma, Cat.no. F2168); Rabbit polyclonal anti γ-Tubulin (Sigma, Cat.no. T5192); Rat anti-human CENP-T (Watanabe et al., 2019, Yoda Lab, Nagoya university); Mouse monoclonal anti-human CENP-A (Obuse et al., 2004, Yoda Lab, Nagoya university); Mouse monoclonal anti-BrdU (BD, Cat.no. 347580); Rabbit anti-Horseradish peroxidase-conjugated (HRP)-conjugated goat anti-rabbit IgG (Jackson ImmunoResearch, Cat.no. 111-035-144); HRP-conjugated goat anti-rat IgG (Jackson ImmunoResearch, Cat.no. 112-035-003); HRP-conjugated rabbit anti-mouse IgG (Jackson ImmunoResearch, Cat.no. 115-035-003); FITC-conjugated goat anti-rabbit IgG F(ab')2 (Jackson ImmunoResearch, Cat.no. 111-095-006); FITC-conjugated goat anti-mouse IgG (Jackson ImmunoResearch, Cat.no. 115-095-003); FITC-conjugated goat anti-rat IgG (Jackson ImmunoResearch, Cat.no. 112-095-003); Cy3-conjugated mouse anti-rabbit IgG (Jackson ImmunoResearch, Cat.no. 211-165-109); Cy3-conjugated goat anti-mouse IgG (Jackson ImmunoResearch, Cat.no. 115-165-003); Alexa647-conjugated goat anti-mouse IgG (Jackson ImmunoResearch, Cat.no. 115-605-003)
Validation	Rabbit polyclonal anti-chicken CENP-T (Hori et al., 2008); Chicken CENP-T antibody was validated using CENP-T conditional KO DT40

Validation

cell line.
Rabbit polyclonal anti-chicken CENP-C (Fukagawa et al., 1999) (in this study); Chicken CENP-C antibody was validated using CENP-C conditional KO DT40 cell line.
Rabbit polyclonal anti-chicken Dsn1 (Hara et al., 2018); Chicken Dsn1 antibody was validated using Dsn1 conditional KO DT40 cell line.
Rabbit polyclonal anti-chicken Nuf2 (Hori et al., 2003); Chicken Nuf2 antibody was validated using Nuf2 conditional KO DT40 cell line.
Rabbit polyclonal anti-chicken Ndc80 (Hori et al., 2003); Chicken Ndc80 antibody was validated using Ndc80 conditional KO DT40 cell line.
Rabbit polyclonal anti-chicken Spc25 (in this study); Chicken Spc25 antibody was validated using Spc25 conditional KO DT40 cell line.
Rabbit polyclonal anti-chicken Knl1 (Hori et al., 2013); Chicken Knl1 antibody was validated using Knl1 conditional KO DT40 cell line.
Rabbit anti polyclonal-chicken Mis12 (Kline et al., 2006); Chicken Mis12 antibody was validated using Mis12 conditional KO DT40 cell line.
Rabbit polyclonal anti-chicken Bub1 (Hori et al., 2013); Chicken Bub1 antibody was validated using DT40 cell and recombinant chicken Bub1 protein.
Rabbit polyclonal anti-chicken Aurora B (Hori et al., 2013); Chicken Aurora B antibody was validated using DT40 cell and recombinant chicken Aurora B protein.
Mouse monoclonal anti-H3T3ph (Hori et al., 2013); H3T3ph antibody was validated in Kimura et al, 2008.
Mouse monoclonal anti-human Ndc80 (abcam, Cat.no. ab3613); Human Ndc80 antibody was validated by abcam using HeLa cell.
Rabbit polyclonal anti-human Dsn1 (Bio Academia, Cat.no. 70-101); Human Dsn1 antibody was validated by Bio Academia using Hela cell and MCF-7 cell.
Rabbit polyclonal anti-GFP (MBL, Cat.no. 598); GFP antibody was validated by MBL using various cell lines expressed.
Mouse monoclonal anti-FLAG M2 (Sigma, Cat.no. F1804); FLAG antibody was validated using DT40 cell lines expressed FLAG-fused protein.
Rat monoclonal anti-RFP (Chromotek, Cat.no. 5f8); RFP antibody was validated by Chromotek using HeLa cell expressed mCherry fused PCNA.
Mouse anti-α-tubulin (Sigma, Cat.no. T9026); α-tubulin antibody was validated by Sigma using chinese hamster embryonic fibroblast cell, chicken fibroblast cell, and human skin fibroblast cell.
FITC-conjugated mouse anti-α-tubulin (Sigma, Cat.no. F2168); α-tubulin antibody was validated by Sigma using chicken fibroblast cell.
Rabbit polyclonal anti γ-Tubulin (Sigma, Cat.no. T5192); γ-tubulin antibody was validated by Sigma using NIH3T3 cell, HeLa cell, and Rat2 cell.
Rat anti-human CENP-T (Watanabe et al., 2019); Human CENP-T antibody was validated using RPE1 cell.
Mouse monoclonal anti-human CENP-A (Obuse et al., 2004); Human CENP-A antibody was validated in Ando et al., 2002 using HeLa cell.
Mouse monoclonal anti-BrdU (BD, Cat.no. 347580); BrdU antibody was validated by BD.

Eukaryotic cell lines

Policy information about cell lines

Cell line source(s)

We used chicken DT40 cell lines and human RPE-1 (ATCC) cell lines : conditional knockout lines, transgene expression lines. The cell lines generated in this study are listed in Supplementary Table 2.

Authentication

Cell lines were examined for their morphology by microscopy and for their protein expression by immunoblot.

Mycoplasma contamination

Cell lines were not tested for mycoplasma contamination.

Commonly misidentified lines
(See ICLAC register)

No commonly used misidentified cell lines were used.

Flow Cytometry

Plots

Confirm that:

☒ The axis labels state the marker and fluorochrome used (e.g. CD4-FITC).

☒ The axis scales are clearly visible. Include numbers along axes only for bottom left plot of group (a 'group' is an analysis of identical markers).

☒ All plots are contour plots with outliers or pseudocolor plots.

☒ A numerical value for number of cells or percentage (with statistics) is provided.

Methodology

Sample preparation

Detailed of sample preparation is described in Methods. In brief, The cells were incubated with BrdU for 20 min at the indicated time after Tet addition and harvested. Following fixation and denaturation, cells were stained with an anti-BrdU antibody and propidium iodide. Cell cycle distribution was analyzed by flow cytometry. Both contour and dot plots are provided in Supplementary table 1.

Instrument

Guava easyCyte (Merck)

Software

guava soft 3.1.1 (Merck), Guava InCyte (Merck)

Cell population abundance	n/a. Flow cytometry was not used for cell sorting, but for cell cycle analysis in this study.
Gating strategy	Gating strategy is provided in Source data. All events were analyzed with FSC-H/SCC-H dot blot (Plot P01, G1). G1 gate was analyzed with Red-B fluorescence (Propidium iodide) Area (Red-B-A/Red-B-H) (Plot P02, G2). G2 gate was analyzed with Green-B fluorescence (BrdU/FITC) and Red-B fluorescence (Propidium iodide) (Plot P03).

☒ Tick this box to confirm that a figure exemplifying the gating strategy is provided in the Supplementary Information.
